# Tannic acid-functionalized 3D porous nanofiber sponge for antibiotic-free wound healing with enhanced hemostasis, antibacterial, and antioxidant properties

**DOI:** 10.1186/s12951-023-01922-2

**Published:** 2023-06-13

**Authors:** Zihang Huang, Donghui Wang, Steffan Møller Sønderskov, Dan Xia, Xiaotong Wu, Chunyong Liang, Mingdong Dong

**Affiliations:** 1 Tianjin Key Laboratory of Materials Laminating Fabrication and Interface Control Technology, School of Materials Science and Engineering, Hebei University of Technology, Tianjin, 300130 China; 2 Center for Health Science and Engineering, School of Health Sciences and Biomedical Engineering, Hebei University of Technology, Tianjin, 300130 China; 3 Interdisciplinary Nanoscience Center (iNANO), Aarhus University, 8000 Aarhus C, Denmark

**Keywords:** 3D nanofiber sponge, Tannic acid, Electrospinning, Wound dressing

## Abstract

Developing an antibiotic-free wound dressing with effective hemostasis and antibacterial and antioxidant capacity is highly desirable. In this work, a three-dimensional (3D) chitosan/polyvinyl alcohol-tannic acid porous nanofiber sponge (3D-TA) was prepared via electrospinning. Compared with two-dimensional (2D) fiber membrane, the unique fluffy 3D-TA nanofiber sponge had high porosity, water absorption and retention ability, hemostatic capacity. Furthermore, the 3D sponge functionalized by tannic acid (TA) endow the sponge with high antibacterial and antioxidant capacity without loading antibiotics. In addition, 3D-TA composite sponges have shown highly biocompatibility against L929 cells. The in vivo experiment shows the 3D-TA is enable to accelerate wound healing. This newly 3D-TA sponges hold great potential as wound dressings for future clinical application.

**Supplementary Information:**

The online version contains supplementary material available at 10.1186/s12951-023-01922-2.

## Introduction

Wound dressings cover damaged skin thereby supplying a temporary healing environment to treat skin wounds. The key factors of these important and widely used medical materials include effective wound sealing to stop bleeding, promotion of blood clotting, prevention of further traumas as well as strong antibacterial and anti-infective capabilities [1, 2]. Many new types of wound dressings, including hydrogels [3, 4], sponges [5], electrospun nanofibers [6, 7], etc., have been developed recently to treat skin deficiency. Due to the similarity to natural extracellular matrix, large specific surface areas and porous structures, electrospun nanofibers provide an ideal biomimicry environment for wound healing [8, 9]. However, due to the highly compact two-dimensional (2D) geometry of traditional nanofiber membranes leading to poor connectivity, low absorptivity, small pore diameter and low porosity, their applications in three dimensions have been limited. Therefore, three-dimensional (3D) nanofibers with hierarchical pore structures have attracted extensive attention because of their improved surface area-to-volume ratio, lower density, better filtration property, superior liquid absorbing capacity, and higher suitability to support cell growth in three dimensions [10–12].

Bacterial infection will trigger body immune response leading to inflammation, tissue damage, and can be potentially life-threatening [13]. Hence, an ideal wound dressing should possess efficient antibacterial properties to avoid serious infection. At present, almost half of commercial wound dressings adopt antibiotics to prevent bacterial infections. However, new problems, including allergy, toxicity and bacterial resistance due to the excessive abuse of antibiotics will arise [14, 15]. In addition, during the inflammatory phase of wound healing, immune cells will secrete pro-inflammatory cytokines, inducing inflammatory cells to produce reactive oxygen species (ROS), including hydrogen peroxide (H_2_O_2_), hydroxyl radical (·OH) and superoxide anion radical [4, 16]. The excessive accumulation of ROS in an infected wound can damage the antioxidant defense system and enhance the inflammatory response, which will delay wound healing and lead to scar formation resulting in skin dysfunction and poor cosmetic appearance [17, 18]. Therefore, it is of vital importance to explore agents having both antibacterial and antioxidant capacities for wound healing.

Natural products of plants are considered to be a source of effective antibacterial agents because of their low toxicities [19]. Tannic acid (TA) based materials have been proved to play a crucial part in biological functions due to their stability in physiological conditions and nontoxicity to cells in vitro [20]. TA is rich in dihydroxy phenyls and the presence of phenolic hydroxyl group gives TA many functions, such as easy binding to other materials through non-covalent interactions, especially hydrogen bonding and ionic coordination, endowing it free radical isolating, anti-inflammatory and antibacterial properties [21–23]. In addition, TA can stimulate blood coagulation through the interaction of its phenolic hydroxyl group with proteins and peptides in blood [24]. These excellent properties make TA an ideal candidate having both antibacterial and antioxidant capacities in wound dressing.

However, the fabrication process of 3D nanofiber sponges will lead to the drugs releasing or inactivated if the drugs are encapsulated in the nanofibers or added before the homogenization treatment [25]. Former studies are either preparing 3D nanofiber scaffolds without drugs or with antibiotics [26]. Study also shows the efficiency of 3D scaffolds prepared by multi-layer electrospinning is relatively low, sometimes stratification even occurs [27]. The pore size of 3D structures prepared by heat-induced phase separation combined with electrostatic spinning is usually small and the porosity is low [28]. Template-assisted collection usually requires a specific collector and complex techniques [29, 30]. 3D layered nanofibers prepared by NaBH_4_ foaming technology involve gas generation in aqueous solution [31], thus is limited to insoluble materials in water. Therefore, the electrostatic spinning and post-processing technology has become an effective simple method to prepare 3D sponges with antibacterial and/or antioxidant agents to resist bacterial infection and oxidation, having no restrictions on materials, and does not require any complex equipment.

Chitosan (CS), a natural polysaccharide biopolymer with good biocompatibility and low toxicity is widely used in the biomedical arena [32, 33]. However, its spinnability is hampered by the repulsive force of ions on the main chain, requiring the co-spinning of other polymers [34, 35]. Polyvinyl alcohol (PVA) has good chemical and physical stability, high hydrophilicity, non-toxicity and biocompatibility [36] and has been approved by the FDA and is widely used in wound dressing. In addition, the hydroxyl group of PVA can bond with the phenolic hydroxyl group of TA *via* a large number of reversible hydrogen bonds, making the sponge with good antibacterial and antioxidant performance. So far, owing to the limitation of the fabrication process, a 3D nanofiber sponge with non-antibiotic single agent having both antibacterial and antioxidant capabilities has rarely been reported.

Herein, a 3D CS/PVA-TA (3D-TA) nanofiber sponge with high porosity, absorptivity, excellent water retention capacity and compression/resilience property was prepared. The grafting of non-antibiotic TA *via* the hydrogen bonding between the PVA and TA endows the 3D-TA sponge with both antibacterial and antioxidant properties. The antibacterial activity, clotting activity and in vitro biocompatibility of the 3D-TA nanofiber sponge were evaluated by plate counting, measuring the hemoglobin content, CCK-8 staining and live/dead assay. The results show that the 3D-TA nanofiber sponges exhibit excellent antibacterial, antioxidant activity and biocompatibility. In addition, in vivo animal experiments show that the 3D-TA sponge could accelerate the healing of full-layer skin defect wounds in mice.

## Materials and methods

### Materials

CS, PVA, tert-butylalcohol and acetic acid (36%) were purchased from Aladdin Reagent Co., Ltd. TA was purchased from Macklin Biochemical Technology Co., Ltd. Anhydrous ethanol was purchased from National Pharmaceutical Co., Ltd. Hemoglobin test solution (C021) and 1,1-Diphenyl-2-picrylhydrazyl radical (DPPH) were obtained from Nanjing JianCheng and glutaraldehyde (2.5%) was purchased from Labcoms. The count assay Kit-8 (CCK-8) was received from Soleibao Technology Co., Ltd. All the reagents and materials were used as received without further treatment.

### Fabrication of different nanofibrous scaffolds

Preparing 2D nanofiber membrane: PVA powder (0.77 g) was added into the deionized (DI) water (7 mL) and stirred at 90 °C until the powder was dissolved. Then, CS (0.09 g) was dissolved in acetic acid solution (3 mL, 36%) and stirred for 4 h for dissolving completely. Finally, the cooled PVA solution is mixed with the CS solution under stirring until both the solutions were mixed completely. For electrospinning, the mixture was encapsulated in a syringe (5 mL) with a needle of 21 gauge. The applied voltage was set as 9 kV with a solution flow rate of 0.04 mm/min. The collecting distance was set as approximately 15 cm from the needle to the collector. The 2D nanofiber membrane was dried for at least 24 h at room temperature (RT) to remove residual solvents.

Preparing 3D nanofiber sponges: 2D nanofibrous mats were cut into fragments (1 cm × 1 cm) and crushed by a homogenizer (FJ200-S, LiChen Technology Co., China) in tert-butylalcohol. The well-dispersed nanofiber mixture was poured into molds froze in the refrigerator for 12 h. The 3D nanofiber sponges were collected after freeze-drying for 24 h. Finally, the 3D samples were cross-linked in 2.5% (v/v) glutaraldehyde vapor for 12 h at RT.

3D nanofiber sponge grafted with TA: The cross-linked 3D samples were immersed in TA solution with concentration of 0.5 mg/mL and 1 mg/mL for 12 h. After washing 3 times with DI water, the 3D sponges (3D-TA_0.5_ and 3D-TA_1.0_) were frozen for 12 h and then freeze-dried for 24 h until the prepared sponges were completely dried.

### Nanostructure characterization

The morphology of different nanofiber scaffolds was observed by scanning electron microscope (SEM, S-4800, Hitachi Co., Japan) with an applied voltage of 15 kV. The samples were sputter-coated with a layer of gold nanoparticles to avoid the surface charge. The diameter of nanofibers was counted by randomly measuring 100 fibers with a Nano Measurer software.

The chemical groups of 3D nanofiber sponges before and after the grafting of TA were characterized by Attenuated Total Reflection Fourier Transform Infrared Spectroscopy (ATR-FTIR, V80, Bruker, Germany). The samples were placed on the sample holder. The spectrum was recorded over the range of 500–4000 cm^− 1^.

The porosity of different scaffolds was evaluated by ethanol displacement method [37]. Briefly, the dry samples were weighed as *w*_*w*_. Then the dry samples were immersed in anhydrous ethanol for 2 h and weighed as *w*_*d*_. The porosity of different scaffolds is calculated according to Eq. 1,

1 $$Porosity \left(\text{\%}\right)=\frac{{w}_{d}-{w}_{w}}{\rho {v}_{0}}\times 100\%$$

where *ρ* and *v*_*0*_ are the density of ethanol and the volume of dry samples, respectively.

### Water absorption rate

Different samples (Control, 2D, 3D, 3D-TA_0.5_, 3D-TA_1.0_) were weighed and placed in DI water for soaking for 1 h at RT. Then the water absorbing rate (*AR*_*water*_) of different samples was evaluated by gravimetric method. The cyclic water absorption performances of 3D nanofiber sponges were designed to compress the cylindrical water-saturated 3D sponges with 80% strain to extrude most of the absorbed water first. After removing the external force, the deformed sponges were then immersed in DI water again. After 20 cycles of absorption and desorption, the *AR*_*water*_ of 3D nanofiber sponges was obtained by Eq. 2,

2 $${AR}_{water} \left(\text{\%}\right)=\frac{{m}_{1}-{m}_{0}}{{m}_{0}}\times 100\%$$

where *m*_*0*_ and *m*_*1*_ are the sample weight before and after absorbing the water.

### Water retention rate

The water retention of Control, 2D, 3D, 3D-TA_0.5_, and 3D-TA_1.0_ was measured by weight method. Briefly, various samples were weighed firstly and put into DI water for 24 h at RT. Then the hydrated samples were taken out and placed in an oven at 37 ℃ and then weighed at preset intervals. The water retention rate (*RR*_*water*_*)* of different supports was calculated according to Eq. 3,

3 $${RR}_{water} \left(\text{\%}\right)=\frac{{w}_{m}-{w}_{0}}{{w}_{1}-{w}_{0}}\times 100\%$$

where *w*_*m*_ is the weight of the sample after it is put into the incubator, while *w*_*0*_ and *w*_*1*_ are the weight of original dry and hydrated samples, respectively.

### Compressibility

The compressibility of 3D nanofiber sponges was characterized by the following steps. Firstly, the original height of each sample was measured. Secondly, the 3D samples were compressed to 80% height of themselves by universal testing machine (HY-0580, Hengyi Co., China). Thirdly, the rebound behavior of 3D nanofiber sponges was recorded after the compression force was removed. Repeating the compression for 20 times to calculate the deformation rate (*DR*) of different samples *via* Eq. 4,

4 $$DR \left(\text{\%}\right)=\frac{{h}_{0}-{h}_{n}}{{h}_{0}}\times 100\%$$

where *h*_*0*_ is the original height of samples while *h*_*n*_ is the spring back height of samples after compression.

### Antioxidant test

DPPH radical scavenging method was utilized to assess the antioxidant activity of 3D-TA. The DPPH solution (39.5 mg/L) was prepared by dissolving DPPH in anhydrous ethanol. Then each sample (3D, 3D-TA_0.5_ and 3D-TA_1.0_) was put into a centrifuge tube (5 mL) and immersed in 3 mL DPPH solution. Then the samples were placed in a dark environment for 1 h. Changes in absorbance at 517 nm were measured using ultraviolet spectrophotometer (U100-star2, Shanghai Insmark Instrument Technology Co., Ltd, China). The DPPH radical scavenging activity (*SA*_*DPPH*_) was calculated according to Eq. 5,

5 $${SA}_{DPPH} \left(\text{\%}\right)=\frac{{A}_{black}-{A}_{sample}}{{A}_{black}}\times 100\%$$

where *A*_*blank*_ and *A*_*sample*_ are the absorbance of DPPH solution and samples mixed with DPPH solution, respectively. Three parallel samples were used for each sample.

### In vitro coagulation test

The control group (gauze) and the experimental group (2D, 3D, 3D-TA_0.5_ and 3D-TA_1.0_) were scissored into discs (8 mm) and then placed in the middle of separated petri dishes. The blood (50 µL containing sodium citrate) was instilled onto different sample surfaces and incubated in an incubator at 37 ℃ for 20 min. After taking out the samples from the incubator, the DI water (25 mL) was further added into the petri dish. Then the coagulation of various dressings was recorded.

The blood clots on different sample surfaces were measured quantitatively by dissolving clots in hemoglobin test solution. The ultraviolet spectrophotometer was used to determine the corresponding absorbance at λ_max_ = 540 nm. The hemoglobin content (*HC*) of each sample was obtained according to Eq. 6,

6 $$HC=(OD-{OD}_{0})\times 367.7$$

where *OD* and *OD*_*0*_ are the absorbance of each sample and the blank, respectively.

The interaction between samples and blood cells was investigated by SEM. Briefly, the samples were soaked in PBS. Then the blood (50 µL) was instilled onto the sample surface. After the samples were placed in a 37 ℃ incubator for 20 min and rinsed with PBS, the blood cells on the samples were fixed with 2.5% glutaraldehyde for 2 h and further dehydration for SEM observation.

The blood absorption capacity (*AC*_*blood*_) was determined by gravimetry. The samples (Control, 2D, 3D, 3D-TA_0.5_ and 3D-TA_1.0_) were weighed and then immersed into the blood for 30 min. After the excessive blood on different sample surfaces was removed with filter paper, and the swollen samples were weighed again. The *AC*_*blood*_ of different samples were calculated according to Eq. 7,

7 $${AC}_{blood} \left(\text{\%}\right) = \frac{{M}_{1}-{M}_{0}}{{M}_{0}}\times 100\text{\%}$$

where *M*_*0*_ and *M*_*1*_ are the weight of the dry sample and the sample with blood, respectively.

### Antibacterial activity

CCK-8 assay: First, Escherichia coli (*E. coli*) and Staphylococcus aureus (*S. aureus*) strains were diluted to 1 × 10^6^ CFU/mL. Then 300 µL of bacterial suspension was instilled to plates containing Control, 2D, 3D, 3D-TA_0.5_ and 3D-TA_1.0_ samples, respectively, with a diameter of 8 mm and incubated at 37 ℃ for 24 h. Next, the samples were delivered to a new plate and incubated for 2 h with CCK-8 assay solution. After removing the CCK-8 detection solution, the corresponding OD value of 100 µL solution was obtained.

Co-culture investigation: Briefly, various samples were co-cultured with *E. coli* and *S. aureus* suspensions (1 × 10^6^ CFU/mL) at 37 ℃ for 24 h. Then the two bacterial solutions were diluted 1 × 10^4^ times and 1 × 10^2^ times and smeared evenly in the solid medium. The number of colonies was recorded accordingly after incubation for 24 h.

SEM observation: The influence of different dressings on the antibacterial effects against *E. coli* and *S. aureus* were observed by SEM. Briefly, the *E. coli* and *S. aureus* (10^6^ CFU/mL) were incubated with various nanofiber dressings at 37 ℃ for 24 h. After rinsing with PBS, the obtained samples were fixed at 4℃ for 6 h with 2.5% glutaraldehyde. The samples were then dehydrated with a series of ethanol with different concentrations (30%, 50%, 70%, 80%, 85%, 90%, 95%, and 100%). Finally, the interaction between the bacteria and various samples were characterized by SEM.

### In vitro cytotoxicity

In order to explore the biocompatibility of different samples, the co-culture of the extract from different samples and cells for 72 h was performed. The extract was prepared according to the national extraction standard of China (ISO 10993-12:2021) when the ratio of material surface area to extraction medium is 3 cm^2^/mL. Firstly 2D, 3D, 3D-TA_0.5_, and 3D-TA_1.0_ were sterilized on both sides by UV for 30 min. Secondly, the sterilized samples were placed in high glucose DMEM medium (Hyclone, USA) containing 1% penicillin/streptomycin (Sigma-Aldrich, Germany) extracting for 24 h. Thirdly, 10% fetal bovine serum (FBS) (PAN, Germany) was added to prepare the material extract for cytotoxicity testing. The fresh cell culture medium (100 µL) containing 5 × 10^3^ L929 cells was added to 96-well plates. After the cells were attached, the extracts of different samples were dropped into 96-well plates with attached cells. Control group was placed in DMEM medium without extract. After 72 h of culture (37 ℃, 5% CO_2_), the cells were stained by Calcein-AM/PI double staining kit for live/dead fluorescence imaging.

CCK-8 method: the extraction preparation was the same as that of co-culture method. The L929 cells (5 × 10^3^) were incubated with the extraction in an, incubator at 37 ℃ with 5% CO_2_ for 1, 3 and 5 days. The cell viability of various samples was assessed by CCK-8 method.

### In vivo wound healing studies

All animal studies were conducted in accordance with the standards of National Code for the Care and Use of Laboratory Animals of China. The mice were given a standard diet and free water during a 12 h light-dark cycle at 25 °C. The wound healing behavior was evaluated by covering different dressings onto the deficient skin of mice. Twelve male BALB/c mice (about 20 g) were randomly divided into 4 groups before surgery. The mice were anesthetized by intraperitoneal injection and then the dorsal region of the mice was shaved with a razor and cleaned with alcohol. A circular full-thickness wound with a diameter of 8 mm was generated on the dorsal area of each mice. Control, 2D, 3D, and 3D-TA_1.0_ dressings were cut into matched sizes, applied to the wound spots and immobilized with Tegaderm. Samples were renewed every 3 days. The accordingly wound healing process was recorded by photographs.

### Histological staining

After the mice were sacrificed at the 15th day, the wound tissues of each group were excised and fixed with formaldehyde (4%) in PBS buffer for 24 h. Then the biopsies were embedded by paraffin and bisected afterwards. After staining with hematoxylin-eosin (H&E) and Masson trichrome, the slices were investigated by an optical microscope.

### Statistics

The quantified results are exhibited as mean ± standard deviation. Student’s t-test is utilized to evaluate differences between groups, where p < 0.05 indicates statistical significance.

## Results and discussion

### Nanostructure of different nanofiber scaffolds

**Fig. 1:**
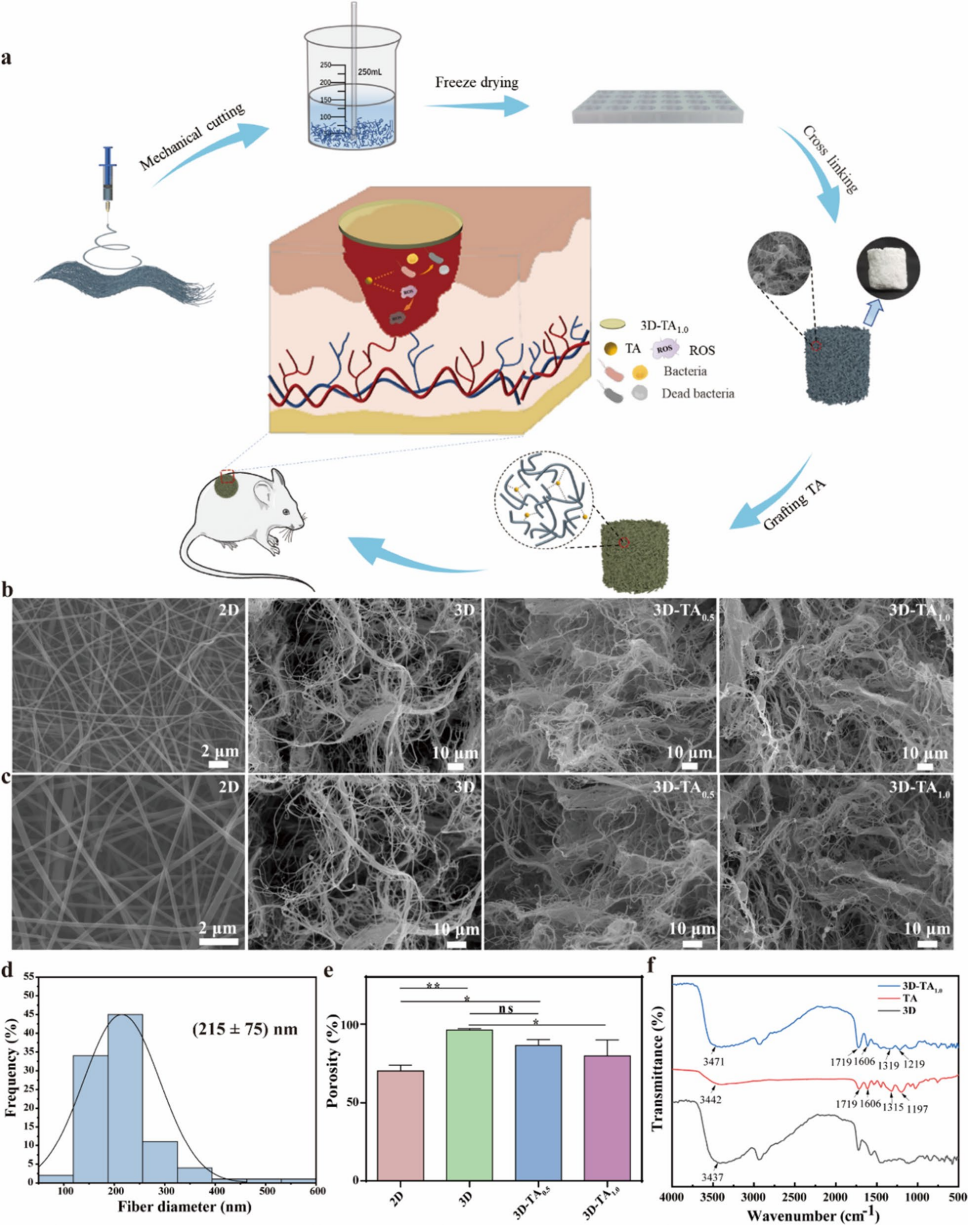
Synthesis and characterization of 3D-TA. **a** Schematics of the preparation of 3D-TA_1.0_ nanofiber sponge. **b**, **c** SEM images of different scaffolds. **d** The fiber diameter distribution of 2D nanofiber membrane. **e** The porosity of different samples. **f** The FTIR spectra of TA, 3D and 3D-TA_1.0_ nanofiber sponges

The preparation of 3D-TA is shown in Fig. 1a. Briefly, 2D nanofiber membranes were fabricated by electrospinning firstly. Then 2D nanofiber membranes were scissored into pieces and homogenized by a homogenizer. Secondly, the homogenized nanofibers were poured into specific molds and freeze-dried for 12 h to prepare 3D nanofiber sponges (3D). The functionalization of 3D nanofiber sponges (3D-TA) was fulfilled by immersing prepared 3D nanofiber sponges into TA solution. The wound healing capacity was verified by observing the wound healing process of circular full-thickness wounds of mice by covering with 3D-TA nanofiber sponges.

The macrostructure of bulk 3D nanofiber sponges is shown in Additional file 1: Fig. S1, where the macrostructure of 3D nanofiber sponges varied along with the fabrication mold and nanofiber sponges with different shapes and heights were prepared. The nanostructure of 2D/3D nanofiber scaffolds is shown in Fig. 1b, c. It is apparent that the 2D nanofiber membrane shows porous structure with smooth nanofiber surface and uniform nanofiber diameter with the distribution of 215 ± 75 nm (Fig. 1d). The morphology of 3D nanofiber scaffolds exhibits a distinct hierarchical spongy structure with large size pores ranging from 32 to 109 μm and a variety of small size pores ranging from 7 to 20 μm. In this hierarchical porous structure, the large pores are formed by sublimation of large ice crystals during freeze-drying of the scaffolds which are thought to facilitate cell ingrowth and cell migration within the scaffold; While the small pores are self-assembled from tangled short fibers which facilitate cell adhesion and proliferation [26]. Meanwhile, the porous structure is also conducive to the entry of nutrients and the elimination of metabolic waste. Figure 1e shows the porosity of different nanofiber scaffolds. The results show the porosity of 2D nanofiber membrane is 70% while that of 3D nanofiber sponge is 96%. This higher porosity makes the 3D nanofiber sponge possess higher specific surface area, enhancing the absorption of wound exudates and the exchange of gas and nutrients effectively. The grafting of different concentrations of TA (3D-TA_0.5_ and 3D-TA_1.0_) did not affect the hierarchical structure of 3D nanofiber scaffold but slightly reduced the porosity and specific surface area. That may be due to the fact that with the increase of TA content on the surface and within pores of nanofiber sponge, more hydrogen bonds are formed between fibers and TA, resulting in adhesion between fibers, thus reducing the porosity and specific surface area of the sponge [38]. However, the porosity of 3D-TA_0.5_ and 3D-TA_1.0_ nanofiber sponge is still higher than that of 2D nanofiber mat with the porosity of 86% and 80%, respectively.

Whether the TA was successfully grafted onto the 3D nanofiber sponges was verified by FTIR (Fig. 1f). The results show that the 3D-TA_1.0_ nanofiber sponge possesses the characteristic peak of carboxylic acid (C = O) vibration at 1719 cm^− 1^, and the benzene ring C = C vibration absorption peak at 1606 cm^− 1^ and benzene ring vibration substitution characteristic peaks at 1219 cm^− 1^. These characteristic peaks were consistent with that of TA indicating the successful grafting of TA onto the 3D nanofiber sponge. Compared with the hydroxyl characteristic absorption peak of 3D (3437 cm^− 1^), the hydroxyl characteristic peak of 3D-TA_1.0_ shifted to the left (3471 cm^− 1^), which attributed to the intermolecular interaction between PVA and TA resulting in the hydrogen bonding [39]. FTIR spectra showed that TA was successfully grafted onto the 3D scaffold by hydrogen bonds.

### Absorption and water retention capacity

**Fig. 2:**
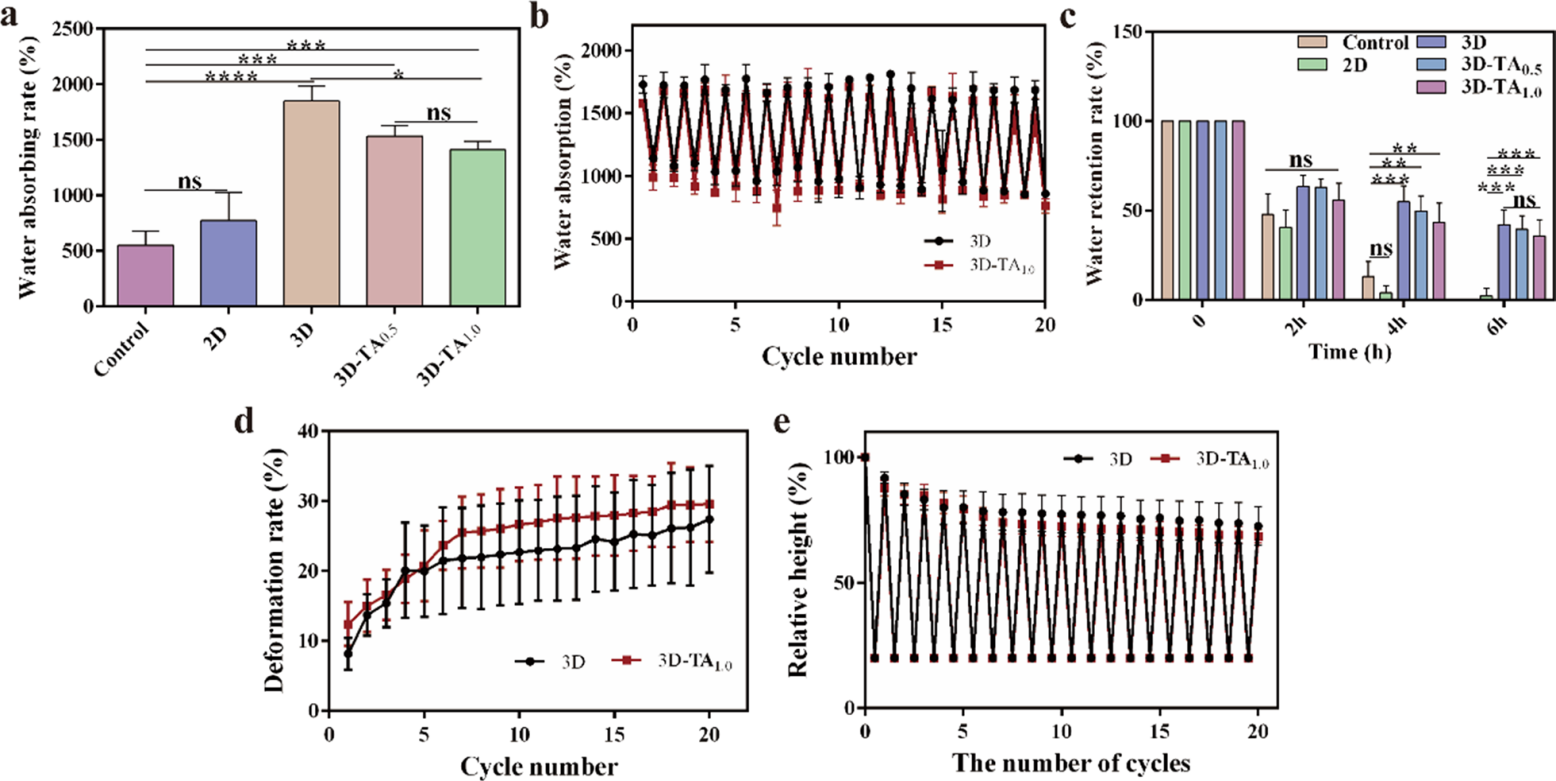
Characterization of nanofiber sponges. **a** The water absorption rates of different samples. **b** Cyclic water absorption/desorption of 3D and 3D-TA_1.0_ nanofiber sponges. **c** The *RR*_*water*_ of different samples within 6 h. **d** The comparison of DR of 3D and 3D-TA_1.0_. **e** The rebound rate of 3D and 3D-TA_1.0_.

Wound dressings should possess good absorption capacity of wound exudates to prevent infection [38]. The water absorption capacities of Control, 2D, 3D, 3D-TA_0.5_ and 3D-TA_1.0_ are shown in Fig. 2a with *AR*_*water*_ values of 551.4% ± 105.7%, 774.2% ± 209.5%, 1848.4% ± 111.6%, 1533.6% ± 77.0% and 1410.0% ± 64.2%, respectively. It is apparent that the absorption ability of 3D sponge is optimal among all the samples (1848.4% ± 111.6%), much higher than that of Control (551.4% ± 105.7%) and 2D nanofiber membrane (774.2% ± 209.5%). That is because the microscale layered porous structure of 3D sponge enhances the water entry and penetration capacity. Moreover, the hierarchical network structure and high porosity of 3D sponge (96%) provide great water storage capacity, making it a good water absorbent. The water absorption of 3D-TA_0.5_ and 3D-TA_1.0_ sponges decreased to 1533.6% ± 77.0% and 1410.0% ± 64.2%, because the grafting of TA decreased the porosity of the 3D sponge (86% and 80%), thus the water absorption of 3D-TA_0.5_ and 3D-TA_1.0_ sponges decreased. However, the hierarchical network structure was not affected after the grafting of TA, therefore the water absorption of 3D-TA_0.5_ and 3D-TA_1.0_ scaffolds was still much higher than that of Control and 2D nanofiber membranes.

The cyclic water absorption capacities of 3D and 3D-TA_1.0_ were verified by a cyclic experiment (Fig. 2b). The results show the 3D and 3D-TA_1.0_ could retain their remarkable water adsorption capacity after repeating twenty adsorption/desorption cycles of water due to the rapid diffusion and infiltration of water into the scaffolds. When the deformed 3D scaffolds are re-immersed in water, the dehydrated samples will quickly and spontaneously absorb water, stimulating the unfolding of the 3D nanofiber sponges caused by compression, resulting in the reversible hygroscopicity of the 3D nanofiber sponges [40].

Wound dressings should not only have good performance of absorbing exudates, but also have the ability to maintain a proper moist microenvironment to promote the regeneration of skin epithelization. The water retention abilities of Control, 2D, 3D, 3D-TA_0.5_ and 3D-TA_1.0_ within 6 h is shown in Fig. 2c. The results show the *RR*_*water*_ of Control and 2D groups decreased to 0 within 6 h while that of 3D, 3D-TA_0.5_ and 3D-TA_1.0_ still maintained 42.0% ± 7.0%, 39.4% ± 6.2%, 35.8519% ± 7.4%, respectively. Again, the water retention ability of 3D is the optimal one. This indicates the 3D, 3D-TA_0.5_ and 3D-TA_1.0_ sponges have good water retention capacity because of the excellent hierarchical porous structure.

### Compression/resilience property

The high elasticity of wound dressing could avoid the wound from being squeezed and deformed by the stress of surrounding tissues or washed away by blood flow when facing a wound with a certain depth [41, 42]. The recovery ability of 3D nanofiber sponges after compression is shown in Additional file 1: Fig. S2, which indicates the rebound height of 3D sponge is lower than the original height. The quantitative analysis of the *DRs* of 3D and 3D-TA_1.0_ after twenty repeated compression/resilience experiments is shown in Fig. 2d. It is found that the *DRs* of both samples increase continuously to ~ 30% during the initial five cycles. That is because the porosity of the 3D samples is large, and the sample density is small. When the compression was applied, the deformation rates of both samples would increase continuously. After five cycles of compression/resilience, both samples were compressed slightly, thus the porosity was reduced and the density of the sample would increase. Therefore, samples would not rebound to the original height (Fig. 2e). In addition, the *DRs* are stable after five cycles of compression/resilience, indicating that the sample height remains unchanged. Comparing the rebound rate of 3D and 3D-TA_1.0_ samples after twenty cycles of compression/resilience, it can be seen that there is little difference between 3D and 3D-TA_1.0_. Both samples can rebound to 75% of its own height after twenty cycles of compression/resilience, which proves that 3D nanofiber sponges possess stable cellular network structure exhibiting good compression/resilience performance.

### Antioxidant activity

**Fig. 3:**
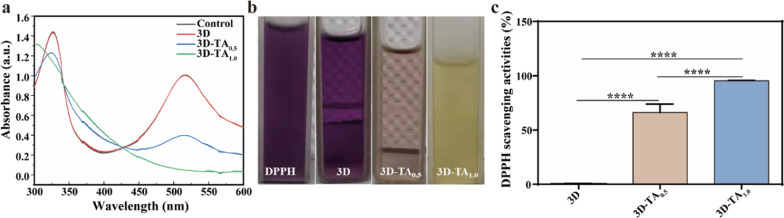
Antioxidant activity. **a** The absorbance of DPPH in different samples. **b** The color change of DPPH blend before and after exposing to 3D, 3D-TA_0.5_ and 3D-TA_1.0_ nanofiber sponges. **c** The *SA*_*DPPH*_ of different samples

The antioxidant activity of wound dressings has been proved to have a positive effect on the wound healing process since it regulates the excessive production of ROS [43]. The antioxidant activities of nanofiber sponges were evaluated by determining their *SA*_*DPPH*_. The absorption plots of DPPH free radical solutions exposed to different samples are shown in Fig. 3a. The absorption curves of 3D and Control group completely coincide indicating the identical intensities. However, the absorption intensities of 3D-TA_0.5_ and 3D-TA_1.0_ are significantly decreased due to the presence of odd number of electrons in DPPH radicals at λ_517_ nm. When electrons are paired with hydrogen atoms in antioxidants, the absorption intensity will reduce, which will result in the color change of DPPH from purple to yellow (Fig. 3b). The degree to which the color becomes lighter is proportional to the number of electrons removed. Figure 3c shows the relative quantified antioxidant properties of three kinds of sponge samples by analyzing the absorbance change at λ_517_ nm with ultraviolet spectrophotometer. It is shown that the *SA*_*DPPH*_ of 3D, 3D-TA_0.5_ and 3D-TA_1.0_ were 0.0%, 66.2 ± 6.4% and 95.5 ± 0.5%, respectively, which indicates that the grafting of TA makes the 3D nanofibers sponge have obvious antioxidant activity and that the higher the TA concentration, the better the antioxidant activity. That is because TA has phenolic hydroxyl, a strong free radical terminator, which enables the reduction the DPPH free radical to the yellow diphenyl picric acid hydrazine, thus achieving the effect of anti-oxidation [39].

### In vitro coagulation performance

**Fig. 4:**
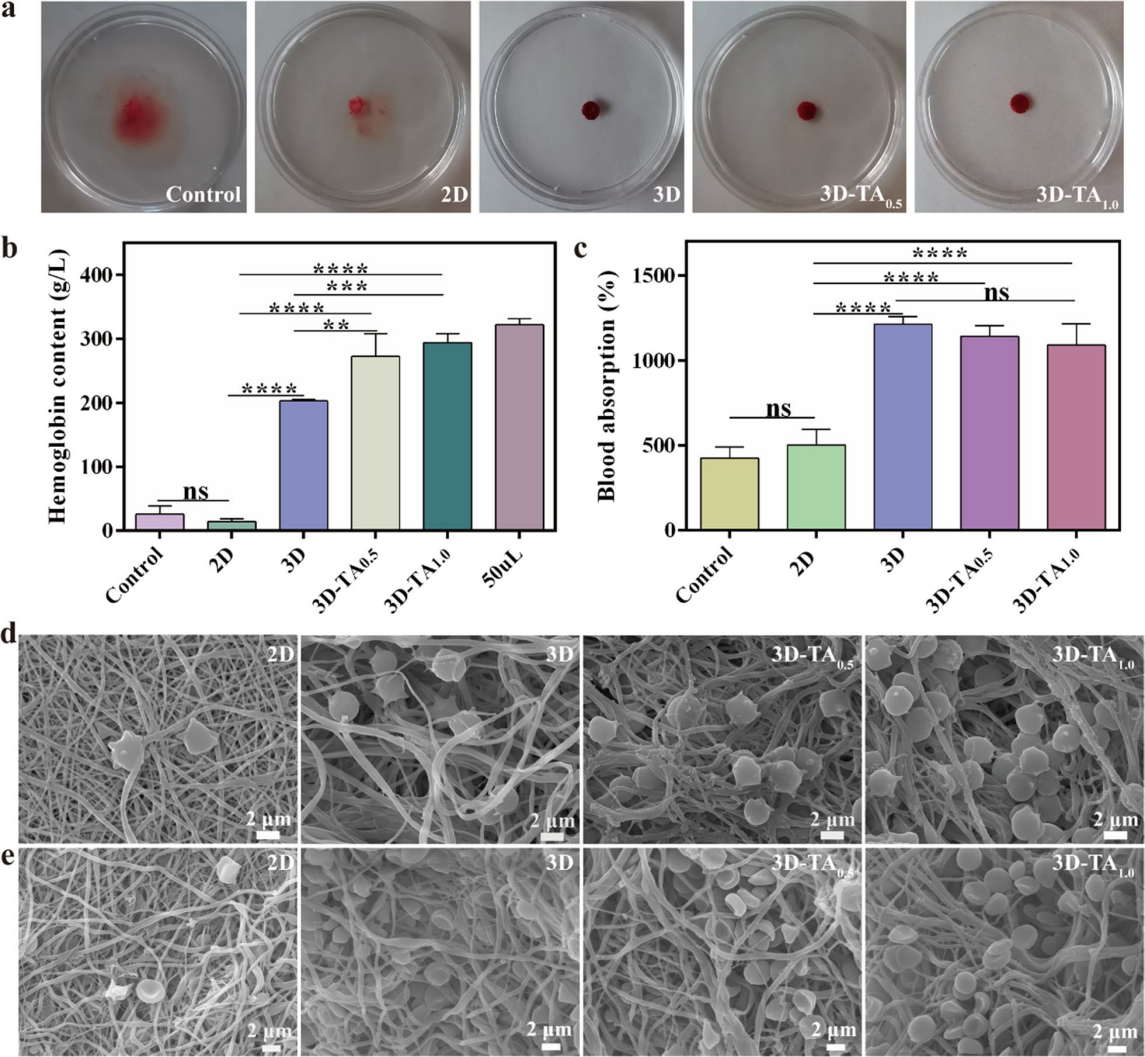
In vitro coagulation performance. **a** The clotting images. **b** *HC* in blood clots and **c** *AC*_*blood*_ of different samples. **d**, **e** SEM images of the interactions between nanofiber surfaces and blood cells; **d**, while blood cells; **e**, red blood cells

The hemostasis ability depends on how effectively the blood clots on the sample. The clotting of Control, 2D, 3D, 3D-TA_0.5_ and 3D-TA_1.0_ samples are shown in Fig. 4a. It is observed that the water in the petri dish turned red for the Control and 2D nanofiber membrane, which indicates that the blood is difficult to clot on medical gauze and 2D fibrous membranes. For 3D, 3D-TA_0.5_ and 3D-TA_1.0_ samples, the blood coagulated well on the samples, displaying transparent water in the petri dish. This indicates that the 3D nanofiber sponges could accelerate blood coagulation when wound is bleeding, and the grafting of TA had no effect on the coagulation effect of 3D nanofiber sponges.

To quantify the hemostatic capacity of various samples, the *HCs* of Control, 2D, 3D, 3D-TA_0.5_ and 3D-TA_1.0_ were measured (Fig. 4b). The *HC* of blood (50 mL) was considered as Control. The higher *HC* of the tested solution indicates its better coagulation effect. The results show the *HCs* of Control, 2D, 3D, 3D-TA_0.5_ and 3D-TA_1.0_ samples were 26.2 ± 10.3 g/L, 14.4 ± 3.4 g/L, 203.2 ± 2.0 g/L, 273. 0 ± 29.1 g/L, 294.4 ± 11.2 g/L, respectively. It is apparent that the *HCs* of 3D, 3D-TA_0.5_ and 3D-TA_1.0_ were much higher than that of Control and 2D samples. Because the abundant porous structure of 3D, 3D-TA_0.5_ and 3D-TA_1.0_ could rapidly soak up blood and inhibit the loss of red blood cells and platelets from the wound, thus significantly enhancing their hemostatic capacity. Besides, the grafting of TA (3D-TA_0.5_ and 3D-TA_1.0_) further improved the clotting ability of 3D fiber sponges. That is because polyphenols can form precipitable complexes with proteins in the blood in a non-specific manner to promote blood clotting [44].

In addition, the *AC*_*blood*_ was also evaluated as shown in Fig. 4c. The Control and 2D nanofiber membrane groups had poor blood absorption capacity with the *AC*_*blood*_ value of 424.8% ± 53.9% and 502.6% ± 74.7%, while the 3D, 3D-TA_0.5_ and 3D-TA_1.0_ had excellent blood absorption capacity with the *AC*_*blood*_ value of 1211.5% ± 38.9%, 1140.6% ± 52.7% and 1089.6% ± 102.9%, respectively. Again, the highly porous structure of the 3D nanofiber sponges accelerated the blood absorption. The addition of TA could affect the porosity of the 3D nanofiber scaffolds, thus would further affect the blood-sucking ability of the 3D nanofiber sponges. However, the difference is not significant.

The interaction between different samples (2D, 3D, 3D-TA_0.5_ and 3D-TA_1.0_) and blood cells was characterized by SEM (Fig. 4d-e). It demonstrates that very few blood cells adhere to the 2D nanofiber membrane, while a large number of blood cells adhere to the 3D, 3D-TA_0.5_ and 3D-TA_1.0_ sponges and most of them immerse into the sample pores, which indicates the hierarchical porous structure of 3D sponges promote the blood coagulation.

### Antibacterial properties

**Fig. 5:**
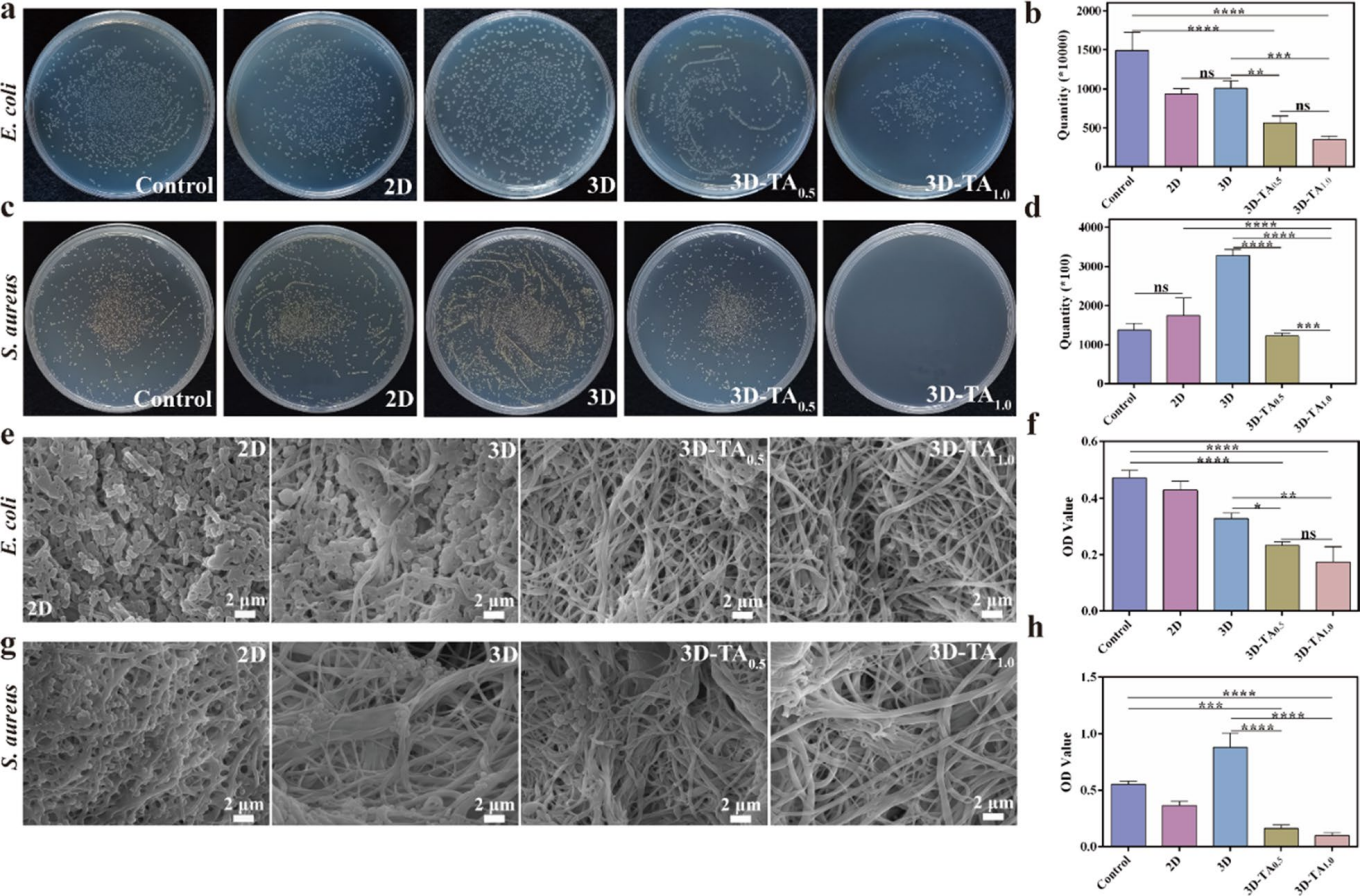
Antibacterial properties. Photographs of colony-forming units of **a ***E. coli* and **c ***S. aureus* on plate after treating with different samples and the corresponding colony count for **b ***E. coli* and **d ***S. aureus*. SEM images of **e ***E. coli* and **g ***S. aureus* on 2D, 3D, 3D-TA_0.5_ and 3D-TA_1.0_ surfaces and the antibacterial ability of different samples against **f ***E. coli* and **h ***S. aureus via* CCK-8 method

Antibacterial ability is an important factor for the clinical application of wound dressing. The antibacterial activities of Control, 2D, 3D, 3D-TA_0.5_ and 3D-TA_1.0_ were determined by plate counting method, SEM observation and CCK-8 method. Figure 5a, c shows the photographs of the colony-forming of *E. coli* (Fig. 5a) and *S. aureus* (Fig. 5c) (10^6^ CFU/mL) on agar plate after being treated with different samples. The corresponding colony count result is shown in Fig. 5b, d. It demonstrates that the scaffolds grafted with TA (3D-TA_0.5_ and 3D-TA_1.0_) have excellent antibacterial performance compared with the Control, 2D and 3D samples. Moreover, the bacteriostatic effect enhances with the increase of TA concentration because TA can inhibit the expression of toxin genes in bacteria inducing cell membrane damage, thus leading to the death of bacterial[45]. In addition, the antibacterial effect of TA on *S. aureus* is higher than that of *E. coli*. Because *E. coli* has an additional lipopolysaccharide cell wall, which is an effective barrier against antibacterial agents and is more resistant to plant extracts [46]. The antibacterial abilities of different samples against *E. coli* (Fig. 5e) and *S. aureus* (Fig. 5g) were further verified by SEM. The results are consistent with that of plate counting method, indicating better antibacterial activity after the grafting of TA onto the 3D nanofiber sponges. Figure 5f, h show the antibacterial ability of different samples against *E. coli* (Fig. 5f) and *S. aureus* (Fig. 5h) *via* CCK-8 detection method. The results show the OD value of 3D-TA_0.5_ and 3D-TA_1.0_ were significantly lower than those of other groups, which again demonstrates the excellent antibacterial ability of 3D nanofiber sponge functionalized with TA.

### Cell viability

**Fig. 6:**
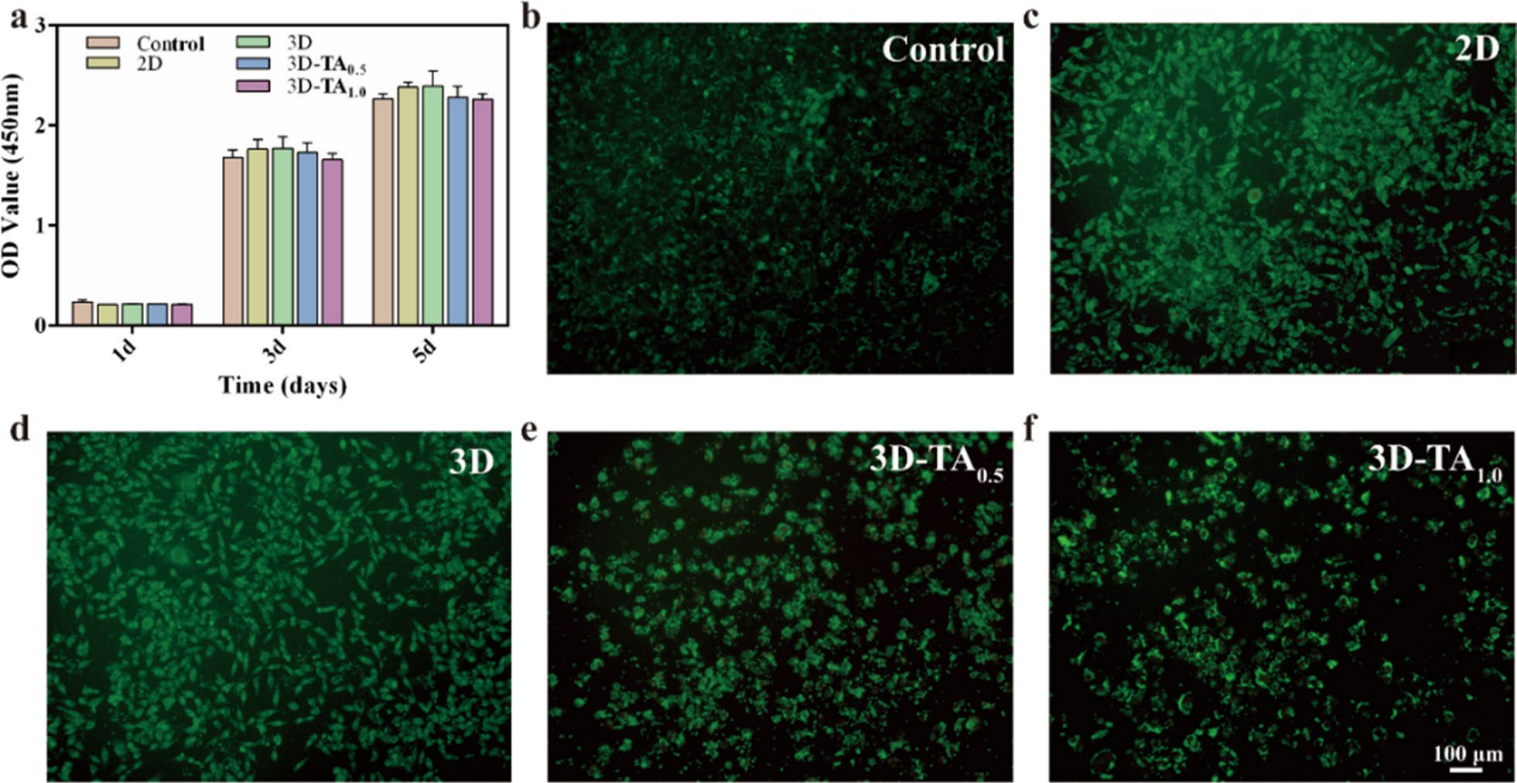
Cell viability. **a** CCK-8 results of L929 co-cultured with different samples extracts for 1, 3, 5 days. **b–f** Live-dead staining fluorescent images of L929 co-cultured with extracts of Control, 2D, 3D, 3D-TA_0.5_ and 3D-TA_1.0_ for 3 days

Wound dressings should be highly biocompatible and promote wound healing through cell proliferation [47]. The cytocompatibility of different dressings was determined by CCK-8 and live/dead staining experiments. Figure 6a shows the CCK-8 results of co-culture cells with extracts of different samples for 1, 3, and 5 days. The Control group was pure medium. It can be seen that the OD values of different samples co-cultured cells with different extracts for 1 day were basically the same as those of the Control with no significant difference, which indicates all samples had good biocompatibility. With the increase of co-culture time, the OD values of all solutions were increased, which proved that the 2D, 3D, 3D-TA_0.5_ and 3D-TA_1.0_ samples had the ability of promoting cell proliferation. Although the OD values of 3D-TA_0.5_ and 3D-TA_1.0_ were slightly lower than those of other groups after co-culturing for 3 and 5 days, there was no significant difference, indicating that the grafting of TA had little effect on cell biocompatibility.

In addition, the live-dead cell experiment was also implemented to verify the good biocompatibility of different samples. Figure 6b–f and Additional file 1: Fig. S3 show the fluorescence images of L929 cells co-cultured with different extracts for 3 days. Here, a large number of living cells (green) and very few dead cells (red) are observed for both the Control and experimental groups (2D, 3D, 3D-TA_0.5_,3D-TA_1.0_) after co-culturing for 72 h. The fluorescence images were consistent with those of CCK-8 results, which demonstrated the good biocompatibility of 3D-TA_1.0_.

### In vivo wound closure ability

**Fig. 7:**
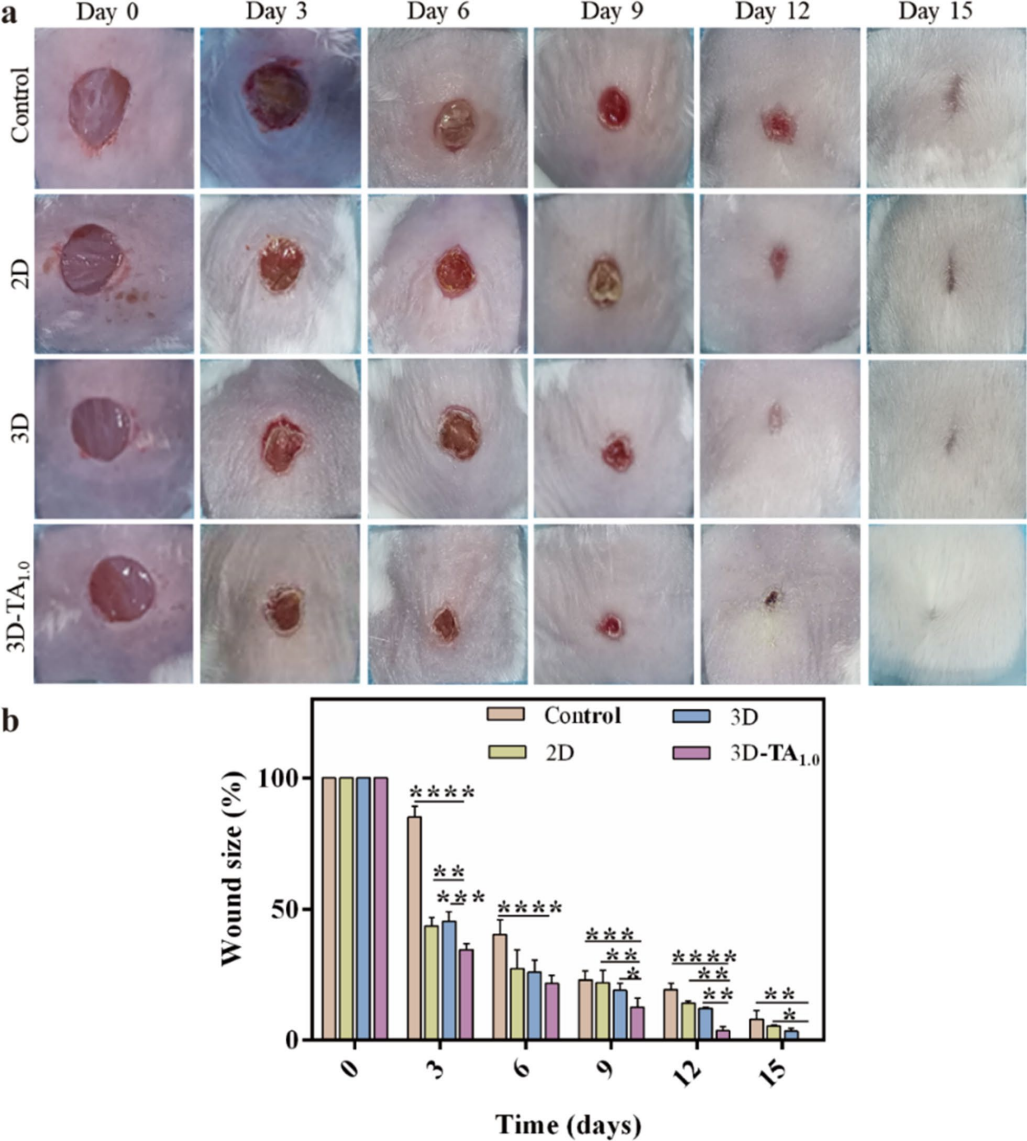
In vivo healing effect. **a** The photographs and **b** wound size of full-thickness wound healing process after treating with Control, 2D, 3D, 3D-TA_0.5_, 3D-TA_1.0_ at various day intervals

To verify the cellular results in vitro, the in vivo wound closure efficiency was evaluated by treating full-thickness wound on the dorsal region of mice with different dressings. The medical gauze was used as Control. Figure 7a shows the optical photographs of wound appearance after treating with different samples (Control, 2D, 3D and 3D-TA_1.0_) at various day intervals (0, 3, 6, 9, 12, 15 days). It is demonstrated that the wound size of all groups decreased with time after treatment. Especially in the 3D-TA_1.0_ group, the reduction of the wound area was the fastest with an almost completely healing at the 15th day while the other three groups covered by Control, 2D and 3D were much larger than that treated with 3D-TA_1.0_. The quantitative analysis of the percentage of wound size over the same time period from the photographs (Fig. 7a) is shown in Fig. 7b. The wound size of 3D-TA_1.0_ group was significantly smaller than that of other groups at all study time points. In the initial stage (day 3), the wound area covered by 3D-TA_1.0_ was reduced to 34.55% ± 1.88% while that covered by Control, 2D and 3D was 85.09% ± 3.48%, 43.59% ± 1.06% and 45.33% ± 2.91%, respectively. On the 9th day, the wound area of Control, 2D, 3D and 3D-TA_1.0_ groups were 23.06% ± 2.79%, 21.95% ± 3.86%, 19.05% ± 2.18% and 12.64% ± 2.74%, respectively. It is also indicated that the wound closure ability of 3D group was better than that of Control and 2D group. At 15 days, the wound in 3D group was basically healed and the wound in 3D-TA_1.0_ group was completely healed without extensive scar and epidermal tissue defect, whereas the obvious wounds were still existed in the other experimental groups. The quantitative results were consistent with the macroscopic observation results, indicating the effective improvement of skin tissue regeneration and promoted cell proliferation capacities of 3D-TA_1.0_.

### Hematoxylin and eosin and Masson’s trichrome

**Fig. 8:**
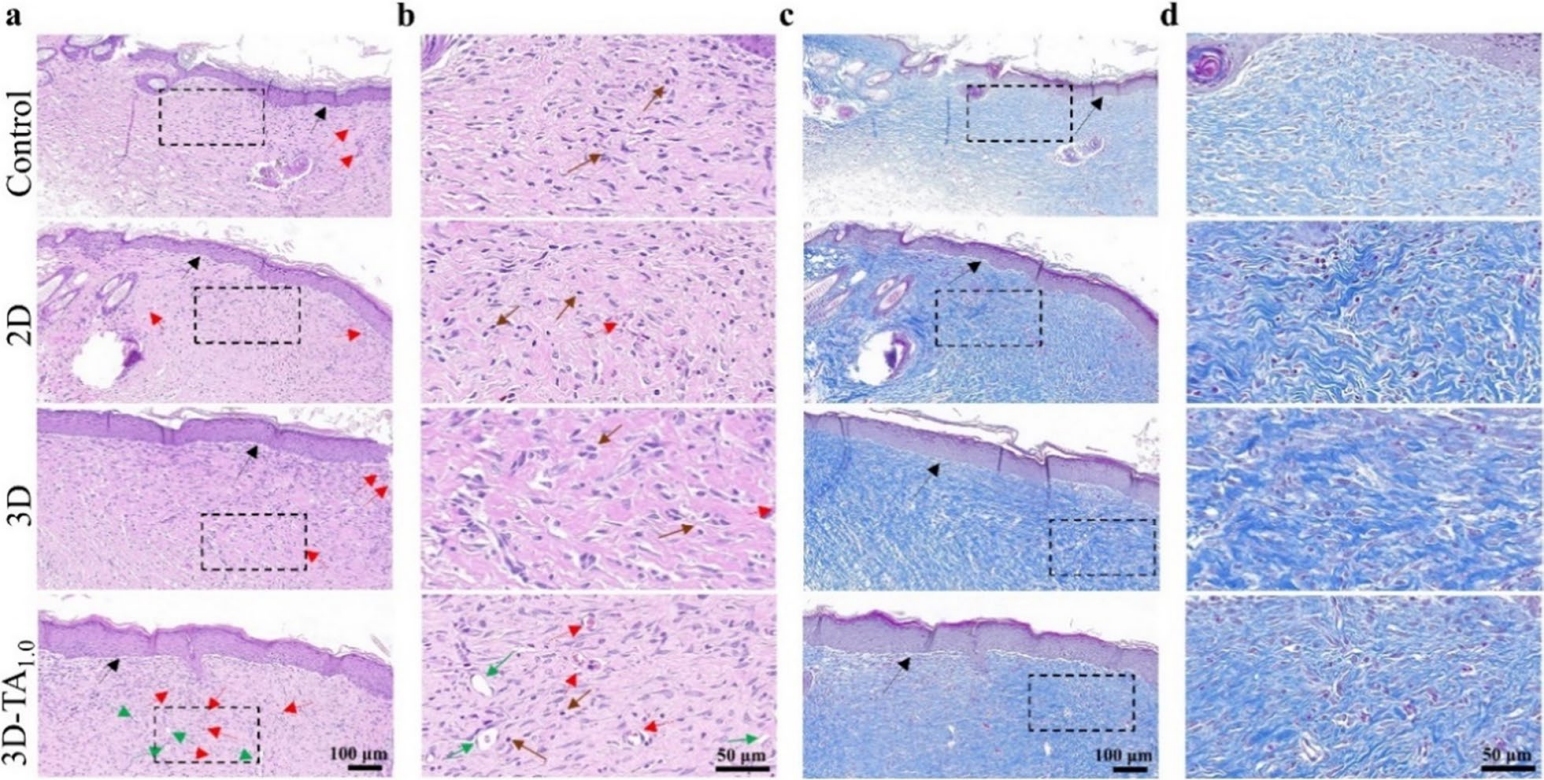
Histological verification of wound healing. **a-b** H&E and **c-d** Masson’s trichrome staining of regenerated cutaneous tissue treated with specified dressings; **b**, **d** The corresponding zoon-in images of black dash squares in **a** and **c**, respectively

To confirm the therapeutic effect of various dressings on wounds, the wound tissues dissected after 15 days of treatment were subjected to hematoxylin and eosin (H&E) and Masson’s trichrome staining (Fig. 8). The H&E staining (Fig. 8a, b) shows that the wound after treatment with 3D-TA_1.0_ underwent more epidermal tissue regeneration (indicated by black arrows) and the presence of fewer inflammation cells (indicated by brown arrows) than that treated with Control, 2D and 3D samples. Moreover, the 3D-TA_1.0_ treated wound exhibited more angiogenesis (indicated by red arrows) and hair follicle cells (indicated by green arrows). The angiogenesis could promote the migration of fibroblasts to the injured site and maintain low levels of inflammation, which eventually leads to a regenerated upper cortex on the wound [48], promoting the wound healing[49]. The Masson trichrome staining (Fig. 8c, d) further verified that more collagen (blue fibrous structure) was deposited after being treated with 3D-TA_1.0_ and the arrangement of collagen fibers was more densely and orderly, similar to normal skin [50].

Overall, the 3D-TA_1.0_ exhibit excellent wound healing ability, which is attributed to the unique hierarchical porous structure of 3D nanofibers sponge facilitating nutrient transport, increasing the air permeability, promoting fibroblast proliferation during wound healing. Moreover, the TA-functionalized 3D sponge endows the 3D-TA sponge with excellent antioxidant and anti-inflammatory properties. The decrease of ROS can reduce inflammatory response, reduce the secretion of pro-inflammatory cytokines, increase the number of macrophages, and trigger the generation of collagen around the wound [51, 52]. In addition, 3D-TA nanofiber sponges further promote angiogenesis and epidermal tissue regeneration, thus accelerate wound healing.

## Conclusion

In conclusion, a 3D-TA_1.0_ nanofiber sponge was prepared *via* electrospinning, which had high porosity, water absorption and retention ability, hemostatic capacity and keep the wound microenvironment moist simultaneously. Moreover, the 3D-TA_1.0_ exhibited excellent antioxidant properties and antibacterial ability. The cellulous experiments showed that 3D-TA_1.0_ had good cell compatibility, which could promote cell adhesion and proliferation. In addition, the promoted wound healing ability of 3D-TA_1.0_ was verified by in vivo experiments. This study indicates that the prepared 3D-TA_1.0_ nanofiber sponge could be an ideal alternative to conventional dressings for future clinical applications.

## Supplementary Information

**Additional file 1: Fig. S1 Macroscopic morphology of 3D nanofiber sponge.** **a** Comparison of 3D nanofiber sponge and 2D nanofiber membrane. The photographsof 3D sponges with different **b** heights and **c** morphologies prepared *via* different molds. **Fig. S2 Compression/resilience process.** The compression/resilience process of 3D and 3D-TA_1.0_ nanofibersponges. **Fig. S3 Cell Live-dead staining.** Live-dead staining florescent images of L929 co-cultured with differentsample extracts for 3 days.

## Data Availability

The data are available within the paper or are available from the authors upon request.
