# Supplementary material for: Tannic acid-functionalized 3D porous nanofiber sponge for antibiotic-free wound healing with enhanced hemostasis, antibacterial, and antioxidant properties
Source: J Nanobiotechnology. 2023 Jun 13;21:190. doi: 10.1186/s12951-023-01922-2 (PMC10262547; doi:10.1186/s12951-023-01922-2)
Supplement: Supplementary file 1 — Additional file 1: Fig. S1 Macroscopic morphology of 3D nanofiber sponge. a Comparison of 3D nanofiber sponge and 2D nanofiber membrane. The photographsof 3D sponges with different b heights and c morphologies prepared via different molds. Fig. S2 Compression/resilience process. The compression/resilience process of 3D and 3D-TA1.0 nanofibersponges. Fig. S3 Cell Live-dead staining. Live-dead staining florescent images of L929 co-cultured with differentsample extracts for 3 days. [file 12951_2023_1922_MOESM1_ESM.docx]

**Additional Materials**

Tannic Acid-Functionalized 3D Porous Nanofiber Sponge for Antibiotic-Free Wound Healing with Enhanced Hemostasis, Antibacterial, and Antioxidant Properties

Zihang Huang^1^, Donghui Wang^2^, Steffan Møller Sønderskov^3^, Dan Xia^1*^, Xiaotong Wu^1^, Chunyong Liang^1, 2*^, Mingdong Dong^3*^

*^1^ Tianjin Key Laboratory of Materials Laminating Fabrication and Interface Control Technology, School of Materials Science and Engineering, Hebei University of Technology, Tianjin 300130, China*

*^2^ Center for Health Science and Engineering, School of Health Sciences and Biomedical Engineering, Hebei University of Technology, Tianjin 300130, China*

*^3^ Interdisciplinary Nanoscience Center (iNANO), Aarhus University, Aarhus C, DK-8000, Denmark*

** Correspondence:* [*xiad@hebut.edu.cn*](mailto:xiad@hebut.edu.cn) *(D.X.);* [*liangchunyong@hebut.edu.cn*](mailto:liangchunyong@hebut.edu.cn) *(C.L.);* [*dong@inano.au.dk*](mailto:dong@inano.au.dk) *(M.D.)*

**Additional file 1: Additional Figures**


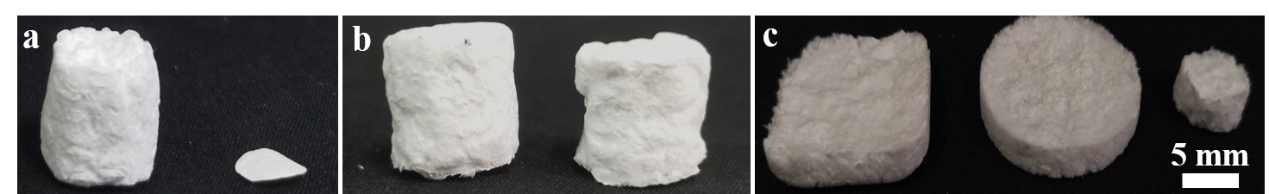


Fig. S1 Macroscopic morphology of 3D nanofiber sponge. (a) Comparison of 3D nanofiber sponge and 2D nanofiber membrane; The photographs of 3D sponges with different (b) heights and (c) morphologies prepared *via* different molds.


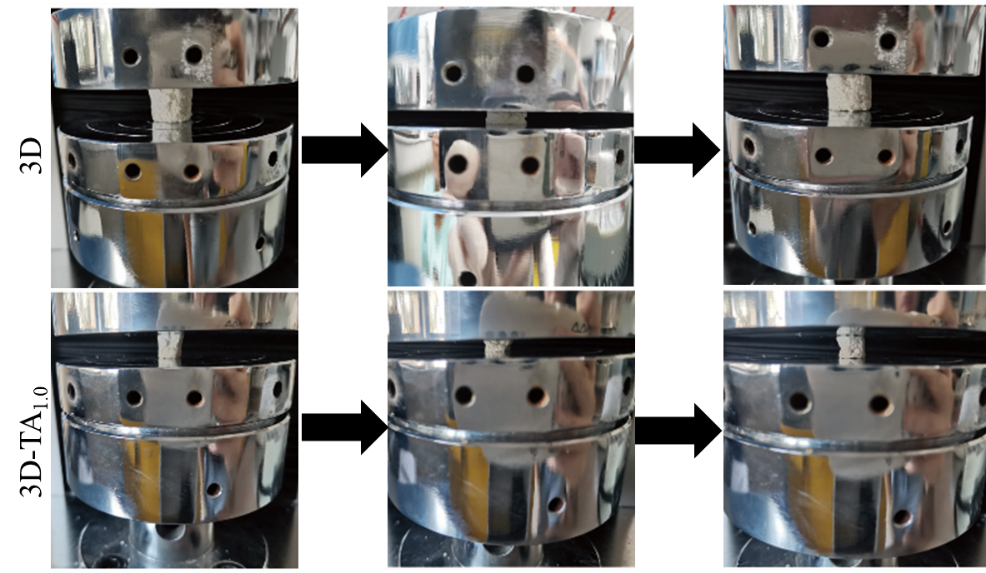


Fig. S2 Compression/resiliecne process. The compression/resiliecne process of 3D and 3D-TA_1.0_ nanofiber sponges.


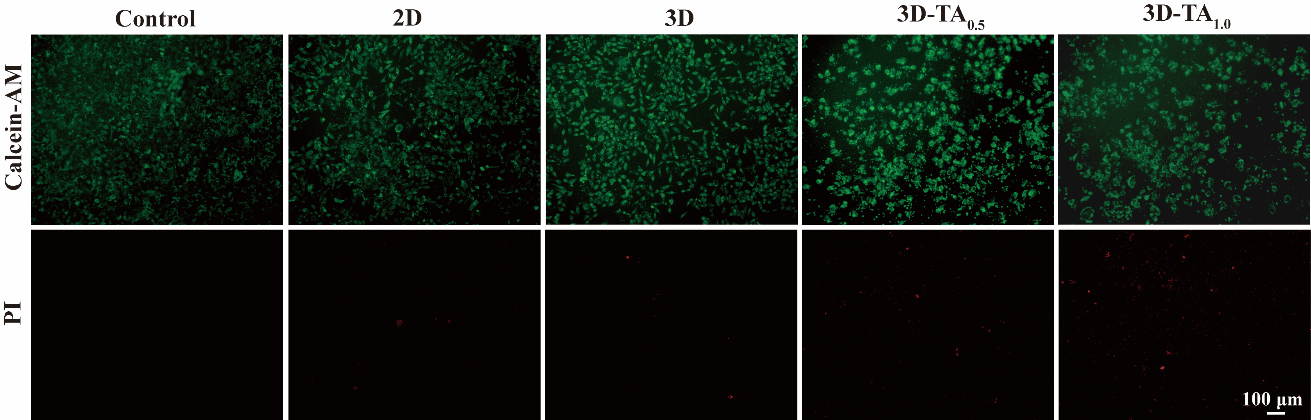


Fig. S3 Cell Live-dead staining. Live-dead staining florescent images of L929 co-cultured with different sample extracts for 3 days.
